# Long-Term Functional and Histological Outcomes Following Sutureless Peripheral Nerve Repair Using Nerve Tape in Pigs

**DOI:** 10.64898/2026.07.29.741584

**Published:** 2026-08-04

**Authors:** Mykhailo M. Tatarchuk, David R. Clizbe, Kevin D. Browne, Susanna Howard, Yohannes Ghenbot, Eric L. Zager, D. Kacy Cullen, Justin C. Burrell

## Abstract

Severe peripheral nerve injuries result in incomplete recovery despite neurorrhaphy. Microsurgical suturing is technically demanding, time-intensive, and may produce variable fascicular alignment. Nerve Tape is an FDA-approved sutureless device enabling rapid, reproducible nerve coaptation. This study compared Nerve Tape with epineurial microsuturing following common peroneal nerve transection in Yucatan minipigs. Over 12 months, both groups demonstrated reinnervation of the tibialis anterior and extensor digitorum brevis, representing proximal and distal muscle targets, respectively. Tibialis anterior recovery was comparable between groups. In contrast, Nerve Tape produced greater distal motor recovery in the extensor digitorum brevis, with approximately 1.8-fold higher compound muscle action potential amplitude and 74.3% versus 46.0% recovery compared with microsutures. Compound nerve action potential amplitudes recorded from the motor branch of the deep peroneal nerve were also greater with Nerve Tape, whereas conduction velocities were comparable. Histological analysis demonstrated preserved fascicular architecture distal to the repair in both groups, with no significant differences in axon count, mean myelinated axon diameter, or g-ratio in the terminal common peroneal nerve or its distal motor branch. Clinical use was demonstrated in a representative case with progressive recovery. Nerve Tape supported durable structural and functional recovery and improved distal motor reinnervation compared with microsuturing.

## Full Text Availability

The license terms selected by the author(s) for this preprint version do not permit archiving in PMC. The full text is available from the preprint server.

